# The impact of outdoor air pollutants on outpatient visits for respiratory diseases during 2012–2016 in Jinan, China

**DOI:** 10.1186/s12931-018-0958-x

**Published:** 2018-12-12

**Authors:** Shanshan Wang, Yifan Li, Aimin Niu, Yao Liu, Lili Su, Wanmei Song, Jinyue Liu, Yunxia Liu, Huaichen Li

**Affiliations:** 1 Department of Public Health, Shandong Provincial Hospital affiliated to Shandong University, Jinan, 250021 Shandong China; 2 Department of Respiratory Medicine, Shandong Provincial Hospital affiliated to Shandong University, Jinan, 250021 Shandong China; 3 Department of Respiratory Medicine, School of Clinical Medicine, Shandong University, Jinan, 250012 Shandong China; 4 Department of Respiratory Medicine, University of Jinan, Jinan, 250022 Shandong China; 5 Department of Biostatistics, School of Public Health, Shandong University, Jinan, 250012 Shandong China

**Keywords:** Air pollution, Outpatient visits, Respiratory diseases, Generalized additive model

## Abstract

**Background:**

Few studies have investigated the associations between outdoor air pollution and outpatient visits for respiratory diseases (RDs) in general population.

**Methods:**

We collected daily outpatient data of primary RDs from five hospitals in Jinan during January 2012 and December 2016, as well as daily measurements of air pollutants from the Jinan Environmental Monitoring Center and daily meteorological variables from the China Meteorological Data Sharing Service System. A generalized additive model (GAM) with quasi-Poisson regression was constructed to estimate the associations between daily average concentrations of outdoor air pollutants (PM_2.5_,PM_10_, SO_2_, NO_2_, CO and O_3_) and daily outpatient visits of RDs after adjusting for long-time trends, seasonality, the “day of the week” effect, and weather conditions. Subgroup analysis stratified by gender, age group and the type of RDs was conducted.

**Results:**

A total of 1,373,658 outpatient visits for RDs were identified. Increases of 10 μg/m^3^ in PM_2.5_, PM_10_, NO_2_, CO and O_3_ were associated with0.168% (95% CI, 0.072–0.265%), 0.149% (95% CI, 0.082–0.215%), 0.527% (95% CI, 0.211–0.843%), 0.013% (95% CI, 0.003–0.023%), and 0.189% (95% CI, 0.032–0.347%) increases in daily outpatient visits for RDs, respectively. PM_2.5_ and PM_10_ showed instant and continuous effects, while NO_2_, CO and O_3_ showed delayed effects on outpatient visits for RDs. In stratification analysis, PM_2.5_ and PM_10_ were associated with acute RDs only.

**Conclusions:**

Exposure to outdoor air pollutants including PM_2.5_, PM_10_, NO_2_, CO and O_3_ associated with increased risk of outpatient visits for RDs.

**Electronic supplementary material:**

The online version of this article (10.1186/s12931-018-0958-x) contains supplementary material, which is available to authorized users.

## Introduction

The adverse effects of outdoor air pollution have attracted intensive attention worldwide. In recent years, China as a heavily air-polluted country has strengthened environmental monitoring, which contributed large amounts of data and provided a unique opportunity to assess the health effects of air pollution [1, 2]. Many recent studies conducted in China have demonstrated that outdoor air pollutants could increase the risk for mortality [3, 4], respiratory diseases (RDs) [5, 6], and cardiovascular diseases [7, 8].

In China, the outdoor air pollution showed a significant regional difference. The composition and levels of pollutants vary from city to city because they are largely depended on meteorological conditions, local terrain and emission sources [9, 10]. For example, the severest air pollution is appear in most cities in north and central China. Moreover, specific populations may be at different risk of air pollution because of the different susceptibility and vulnerability to adverse effects of pollutants [11]. For these reasons, the effect size of air pollution of one region cannot reflect that of another.

Jinan, a capital city located in the east of China, has been experiencing serious air pollution which poses huge threats to people’s health of respiratory system. Previous studies conducted in Jinan have shown that exposure to air pollution is associated with overall-mortality and cause-specific mortality [12], hospital emergency room visits for RDs [13], and acute exacerbations of chronic obstructive pulmonary disease hospitalization [14]. It is noteworthy that these previous studies have been focused on severe cases of RDs, which usually occur in a small proportion of the population in very poor health. The effects of air pollution on the majority of the population remain largely unknown. The outpatient services is open to all diseases with various severity and is not restricted by bed availability, thus could reflect the medical need of most people. Therefore, the effects of air pollution on outpatient visits for RDs need to be explored in large-scale studies.

In the present study, we investigated the associations between outdoor air pollutants and outpatient visits for RDs in Jinan. To do so, we used data from approximately 1.37 million outpatient records in five hospitals between 2012 and 2016.

## Methods

### Data collection

The daily count of outpatient visits for primary RDs from January 1, 2012, to December 31, 2016 were obtained from five hospitals in Jinan city, including Shandong Provincial Hospital Affiliated to Shandong University, Qilu Children’s Hospital of Shandong University, Jinan Central Hospital, Qianfo Mountain Hospital and The Second Hospital of Shandong University. The medical records include patients’ name, age, gender, date of hospital visiting and all diagnoses. We collected data on four main RDs, including pneumonia, acute bronchitis, asthma and chronic bronchitis. Other diagnoses such as interstitial pneumonia, aspiration pneumonia and endogenous lipid pneumonia were excluded because they were few in number.

Data on daily 24-h mean concentrations of PM_10_, PM_2.5_, SO_2_, NO_2_, CO and O_3_ recorded by 14 monitoring stations were obtained from the Environmental Monitoring Center of Jinan. The daily concentration of each pollutant was calculated as the mean of the concentrations recorded by 14 monitoring stations. Besides, daily temperature, humidity, air pressure and wind speed during the study period were downloaded from the China Meteorological Data Sharing Service System (https://data.cma.cn/en).

### Statistical analysis

In descriptive analysis, mean, standard deviation (SD), minimum (Min), maximum (Max), 25th percentile (P_25_), 50th percentile (P_50_) and 75th percentile (P_75_) were used to describe the data on air pollutants, meteorological parameters and outpatient visits. Spearman correlation was used to examine the relationships between air pollutants and meteorology parameters.

Generalized additive model (GAM) is a flexible and effective technique for estimating the unknown non-linear relationship between health effects and air pollution [15, 16]. It does not require the shape of the response curve as a priori knowledge, and allows for nonparametric adjustments for nonlinear confounding effects [17]. Since the outpatient visits typically follow a quasi-Poisson distribution [16, 18], GAM with quasi-Poisson regression were constructed to examine the associations between air pollution and RDs outpatient visits. A penalized smoothing spline function [19] was used to adjust for long-term trends and seasonality in daily outpatient visits and potential non-linear effects of meteorological factors. Specifically, we used 7 degrees of freedom (df) per year for calendar time, and 4 df for mean temperature, relative humidity, air pressure and wind speed according to the Akaike’s information criterion (AIC) [20]. A smaller AIC value indicates a better fitting model [16]. In addition, the model was adjusted for day of the week (DOW) to control the day-in-week of the outpatient visits. Briefly, the following model was fitted:

$$ \mathrm{logE}\left(\mathrm{Yt}\right)=\mathrm{Intercept}+\upbeta \mathrm{Zt}+\mathrm{s}\left(\mathrm{time},\mathrm{df}=7\right)+\mathrm{DOW}+\mathrm{s}\left(\mathrm{temperature},\mathrm{df}=4\right)+\mathrm{s}\left(\mathrm{humidity},\mathrm{df}=4\right)+\mathrm{s}\left(\mathrm{pressure},\mathrm{df}=4\right)+\mathrm{s}\left(\mathrm{wind}\ \mathrm{speed},\mathrm{df}=4\right) $$

where E(Yt) is expected number of daily outpatient visits for RDs on day t; β is the regression coefficient; Zt is the daily concentration of air pollutant on day t; s() denotes the smoother based on the penalized smoothing spline; DOW is the day of the week as a categorical variable.

In the present study, single-pollutant models were firstly fitted for PM_2.5_, PM_10_, SO_2_, NO_2_, CO or O_3_ on the same day and up to 5 days (lag0, lag1, lag2, lag3, lag4 and lag5) and moving averages of 2-day, 3-day, 4-day, 5-day and 6-day (lag01, lag02, lag03, lag04 and lag05). The lagged effects and cumulative effects of each pollutant on RDs outpatient visits were calculated. In addition, we developed two-pollutant models [17] and conducted multicollinearity diagnosis. For two pollutants without obvious collinearity, two-pollutant models were constructed on the lag day with the maximum effect estimates adding the other air pollutant to check whether the associations were still significant. Subgroup analyses were conducted according to gender (male and female), age group (< 18 years, 18–44 years, 45–64 years and > 64 years), and the type of RDs (acute RDs and chronic RDs). Pneumonia and acute bronchitis were defined as acute RDs. Asthma and chronic bronchitis were defined as chronic RDs. All results were expressed as the percentage changes in daily outpatient visits and its 95% CIs associated with a 10 μg/m^3^ increase in air pollutants [18]. All statistical analyses were conducted using R software version 3.4.4 (https://www.r-project.org). The “mgcv” package was used to fit the GAM model. In all analyses, *P* values < 0.05 were considered statistically significant.

## Results

Table 1 summarizes the basic descriptive information of the daily air pollutants, meteorological parameters and outpatient visits. During the study period, the mean pollutant concentrations were 93.5 μg/m^3^ for PM_2.5_, 166.1 μg/m^3^ for PM_10_, 68.8 μg/m^3^ for SO_2_, 52.9 μg/m^3^ for NO_2_, 1384 μg/m^3^ for CO, and 101.3 μg/m^3^ for O_3_. The mean temperature, humidity, pressure and wind speed were 15 °C, 56.3%, 996.5 kPa and 2.5 m/s, respectively. A total of 1,373,658 outpatient visits for RDs were identified, with an average of 752 outpatients per day. The majority of the outpatients were male, less than 45 years old and diagnosed with acute bronchitis.

**Table 1:** Descriptive statistics on daily air pollutants, meteorological parameters and outpatient visits in Jinan, China, 2012–2016

Variable	Mean ± SD	Min	P_25_	P_50_	P_75_	Max
Pollutants (μg/m^3^)						
PM_2.5_	93.47 ± 56.37	14.90	56.00	80.00	113.00	443.00
PM_10_	166.05 ± 79.85	29.10	113.00	150.00	202.50	693.00
SO_2_	68.75 ± 50.30	12.00	36.00	52.00	86.00	429.00
NO_2_	52.94 ± 21.12	13.00	38.00	49.00	64.00	165.00
CO	138.40 ± 640.23	445.00	975.50	1221.00	1590.00	6555.00
O_3_	101.32 ± 59.03	9.90	53.00	89.00	143.00	285.00
Meteorological parameters						
Temperature (°C)	15.04 ± 10.50	−12.40	5.50	17.10	24.10	34.00
Humidity (%)	56.26 ± 19.43	13.00	41.00	55.00	70.00	100.00
Pressure (kPa)	996.52 ± 9.11	975.70	988.70	996.60	1003.70	1021.80
Wind speed (m/s)	2.47 ± 1.07	0.20	1.70	2.20	3.00	8.40
Outpatient visits (cases/per day)						
Total	752.00 ± 250.00	349.00	580.00	680.00	828.00	1631.00
Male	457.00 ± 153.00	210.00	353.00	413.00	503.00	1021.00
Female	294.00 ± 100.00	120.00	225.00	267.00	327.00	643.00
Age						
<18y	281.90 ± 116.25	128.00	204.00	240.00	321.50	780.00
18-44y	260.80 ± 96.26	88.00	191.00	235.00	311.00	593.00
45-64y	120.50 ± 39.30	38.00	93.00	116.00	141.00	282.00
>64y	88.62 ± 38.32	18.00	59.00	83.00	111.00	276.00
Diagnosis						
Acute bronchitis	506.00 ± 179.83	211.00	380.00	458.00	568.50	1152.00
Pneumonia	195.87 ± 80.36	65.00	140.00	169.00	232.00	482.00
Asthma	27.45 ± 15.57	2.00	17.00	24.00	35.00	113.00
Chronic bronchitis	22.20 ± 15.10	0.00	11.00	20.00	30.00	97.00

*SD* Standard deviation, *Min* minimum, *Max* Maximum, *P*_*25*_ 25th percentile, *P*_*50*_ 50th percentile, *P*_*75*_ 75th percentile;

The spearman correlation coefficients between air pollutants and meteorological parameters are shown in Table 2. Each of the meteorological parameter significantly correlated with air pollutants. Temperature negatively and pressure positively correlated with the concentrations of PM_2.5_, PM_10_, SO_2_, NO_2_, and CO. Besides, the air pollutants were significantly correlated with each other. These results indicated that the confounding effects of the meteorological parameters and the effects of other air pollutants should be controlled in models.

**Table 2:** Spearman correlation coefficients between air pollutants and meteorological parameters in Jinan, China, 2012–2016

	PM_2.5_	PM_10_	SO_2_	NO_2_	CO	O_3_	Temperature	Humidity	Pressure	Wind speed
PM_2.5_	1	0.863^**^	0.606^**^	0.642^**^	0.781^**^	−0.182^**^	−0.269^**^	0.131^**^	0.238^**^	−0.236^**^
PM_10_		1	0.613^**^	0.683^**^	0.670^**^	−0.125^**^	−0.235^**^	−0.116^**^	0.223^**^	−0.127^**^
SO_2_			1	0.702^**^	0.674^**^	− 0.344^**^	− 0.588^**^	− 0.293^**^	0.532^**^	− 0.036
NO_2_				1	0.782^**^	−0.459^**^	−0.524^**^	− 0.069^**^	0.557^**^	− 0.391^**^
CO					1	−0.435^**^	−0.455^**^	0.172^**^	0.414^**^	−0.385^**^
O_3_						1	0.794^**^	−0.099^**^	− 0.679^**^	0.199^**^
Temperature							1	0.179^**^	−0.885^**^	0.067^**^
Humidity								1	−0.239^**^	−0.354^**^
Pressure									1	−0.119^**^
Wind speed										1

^**^P < 0.01

**Fig. 1:**
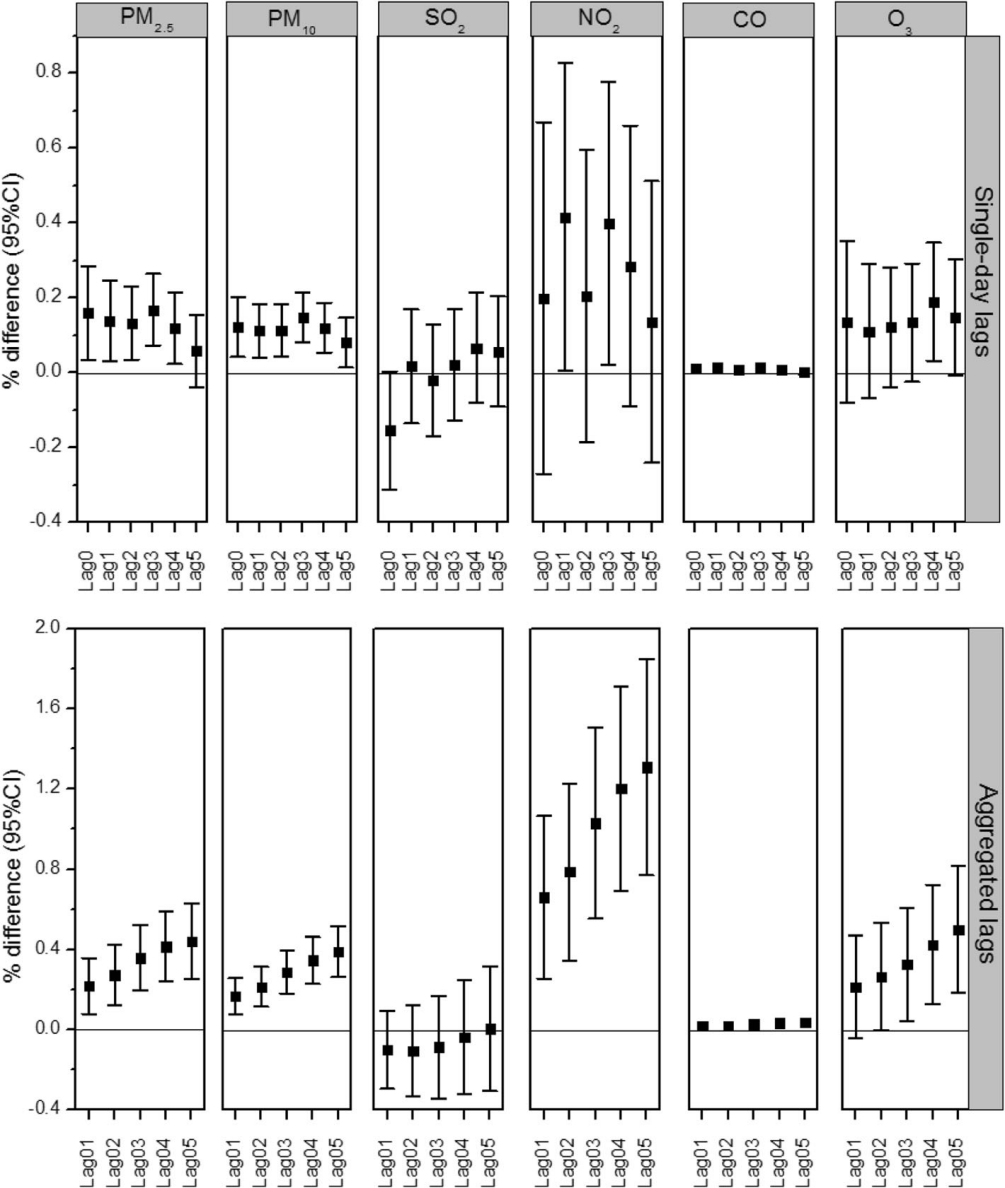
Percentage changes in outpatient visits for RDs associated with a 10 μg/m^3^ increase in concentration of each pollutant

Figure 1 (with detailed data in Additional file 1: Table S1) shows the percentage changes in outpatient visits for RDs associated with a 10 μg/m^3^ increase in concentration of each pollutant for different lag structures. In single-day lags models, an increase of 10 μg/m^3^ of PM_2.5_ and PM_10_ was associated with an increase of 0.159% (95% CI, 0.035–0.284%) and 0.122% (95% CI, 0.042–0.202%) in outpatient visits in the concurrent day, respectively. PM_2.5_ and PM_10_ showed similar lag patterns in association with outpatient visits for RDs. The effect estimates of PM_2.5_ and PM_10_ concentrations increased from lag day 0 to 3, and peaked at lag day 3, then decreased to lag day 5. The maximum estimates for the effects of PM_2.5_ and PM_10_ were 0.168% (95% CI, 0.072–0.265%) and 0.149% (95% CI, 0.082–0.215%), respectively. Additionally, NO_2_, CO and O_3_ were also associated with outpatient visits, the maximum estimates for the effects of were 0.527% (95% CI, 0.211–0.843%), 0.013% (95% CI, 0.003–0.023%), and 0.189% (95% CI, 0.032–0.347%), respectively. SO_2_ was not associated with outpatient visits. In aggregated lags models, the pooled effect estimates from lag day 0 to 5 for PM_2.5_ and PM_10_ were 0.441% (95% CI, 0.251–0.632%) and 0.388% (95% CI, 0.261–0.514%), respectively.

Table 3 compares the maximum effect estimates of air pollutants using single-pollutant models and two-pollutant models. The effect estimates of PM_2.5_ were still significant after adding SO_2_, CO and O_3_ in two-pollutant models. However, the effect of PM_2.5_ lose its statistical significance after adjusting for NO_2_. After adding other air pollutants, the effects for PM_10_ remained significant, indicating that the association between PM_10_ and outpatient visits for RDs was robust. For SO_2_, when PM_2.5_, NO_2_ and CO were introduced separately, a negative association with outpatient visits was observed.

**Table 3:** The maximum effect estimates of air pollutants using single-pollutant models and two-pollutant models

Lag day	Single-pollutant model	Two-pollutant model	Two-pollutant model	Two-pollutant model	Two-pollutant model	Two-pollutant model
PM_2.5_	PM_2.5_	PM_2.5_+ SO_2_	PM_2.5_ + NO_2_	PM_2.5_ + CO	PM_2.5_ + O_3_	
Lag 3	0.168(0.072, 0.265)*	0.217(0.105,0.328)*	0.094(−0.042,0.231)	0.183(0.001,0.366)*	0.168(0.072,0.265)*	
Lag05	0.441(0.251, 0.632) *	0.566(0.343,0.770)*	0.235(−0.024,0.495)	0.481(0.127,0.836)*	0.423(0.233,0.613)*	
PM_10_	PM_10_	PM_10_ + SO_2_	PM_10_+ NO_2_	PM_10_ + CO	PM_10_+ O_3_	
Lag 3	0.149(0.082,0.215)*	0.199(0.121,0.277)*	0.133(0.037,0.230)*	0.197(0.088,0.307)*	0.146(0.079,0.212)*	
Lag05	0.388(0.261,0.514)*	0.501(0.357,0.645)*	0.339(0.159,0.520)*	0.477(0.284,0.669)*	0.368(0.240,0.496)*	
SO_2_	SO_2_	SO_2_ + PM_2.5_	SO_2_+ NO_2_	SO_2_ + CO	SO_2_+ O_3_	SO_2_ + PM_10_
Lag 4	0.066(−0.082,0.214)	−0.038(−0.210,0.134)	− 0.198(− 0.412,0.017)	−0.041(− 0.226,0.144)	0.080(− 0.068,0.229)	−0.106(− 0.280,0.068)
Lag05	0.004(− 0.308,0.316)	−0.410(− 0.756,-0.063)*	−1.091(−1.527,-0.653)*	−0.420(− 0.783,-0.056)*	0.014(− 0.297,0.325)	−0.578(− 0.926,-0.229)
NO_2_	NO_2_	NO_2_+ PM_2.5_	NO_2_+ SO_2_	NO_2_ + CO	NO_2_+ O_3_	NO_2_ + PM_10_
Lag 1	0.527(0.211,0.843)*	0.459(0.034,0.885)*	0.995(0.550, 1.442)*	0.516(0.005, 1.030)*	0.563(0.245,0.882)*	0.361(−0.080,0.805)
Lag05	1.310(0.770,1.852)*	0.853(0.119,1.592)*	2.689(1.911,3.473)*	1.225(0.429,2.027)*	1.336(0.798,1.877)*	0.286(−0.471,1.049)
CO	CO	CO+ PM_2.5_	CO+ SO_2_	CO + NO_2_	CO+ O_3_	CO + PM_10_
Lag 1	0.013(0.003,0.023)*	0.0008(−0.009,0.025)	0.019(0.007, 0.031)*	0.000(−0.016,0.016)	0.014(0.004, 0.024)*	0.003(−0.013,0.019)
Lag05	0.034(0.016,0.051)*	−0.004(− 0.038,0.029)	0.046(0.025,0.067)*	0.004(− 0.023,0.030)	0.038(0.020, 0.056)*	− 0.016(− 0.043,0.010)
O_3_	O_3_	O_3_+ PM_2.5_	O_3_+ SO_2_	O_3_ + CO	O_3_+ NO_2_	O_3_ + PM_10_
Lag4	0.189(0.031,0.347)*	0.188(0.031,0.346)*	0.196(0.038,0.354)*	0.226(0.066,0.387)*	0.221(0.063,0.380)*	0.171(0.014,0.329)*
Lag05	0.499(0.183,0.815)*	0.452(0.138,0.767)*	0.499(0.183,0.816)*	0.593(0.276,0.911)*	0.522(0.208,0.837)*	0.383(0.068,0.699)*

*P < 0.01

Additional file 1: Table S2 and Table S3 presents the effect estimates of PM_2.5_ and PM_10_ across different gender and age groups. The maximum effect estimates of PM_2.5_ and PM_10_ appeared at lay day 3 in both males and females. Among 4 age groups, the maximum effect estimate of PM_2.5_ and PM_10_ was observed for outpatients older than 64 years (at lay day 3) and for outpatients aged between 45 and 64 years (at lay day 4), respectively.

Additional file 1: Table S4 compares the associations of PM with two types of RDs. PM_2.5_ and PM_10_ were associated with acute RDs only. The maximum effect estimates were observed at lay day 3. No significant association of PM_2.5_ or PM_10_ was observed for chronic RDs.

## Discussion

In the present study, we found that outdoor air pollutants (PM_2.5_, PM_10_, NO_2_, CO and O_3_) were associated with increases in daily outpatient visits for RDs. Most of the associations remained significant after using different lag structure or adjusting for other pollutants. In single-day lag model, the effect estimates of PM_2.5_ and PM_10_ peaked at lay day 3. Significant effect modification by the type of RDs were observed. PM_2.5_ and PM_10_ were significantly associated with acute RDs including pneumonia and acute bronchitis. No significant associations were observed for chronic RDs.

The studies on air pollution and outpatient visits mainly conducted in China, because hospital health service is accessible to all patients and usually first-come first-served [21]. Previous studies focused on different air pollutants and different RDs. To a large extent, our results were consistent with previous studies [18, 22–24]. For example, a recent case-crossover study in Beijing, found that PM_2.5_, PM_10_, NO_2_ and CO were positively associated with outpatient visits for four kinds of acute respiratory outcomes [24]. A time-series analysis in Shanghai showed that the effect estimates for NO_2_ were the greatest in magnitude in association with various RDs; additionally, air pollutants were not associated with chronic RDs such as asthma and chronic obstructive pulmonary disease [18]. In this study, we found that exposure to PM_2.5_ and PM_10_ had significantly instant effects on outpatient visits, and the effects lasted for 5–6 days and peaked at 3 days later. Previous studies have been found that PM had instant effect [25], lag effect [26, 27] or both effects [28] on outpatient visits for RDs. The difference of these conclusions might due to study population, sample size, or statistical methods.

The mechanisms linking exposure to PM and potential health consequences have been widely studied. PM_10_, especially PM_2.5_, can be inhaled deeply into the human lung, which attribute to PM a high toxicity. The inflammatory response is considered to be a key point to understand the pathogenesis of diseases [29]. PM_2.5_ exposure has been associated with various inflammatory biomarkers, including exhaled nitric oxide level [30, 31], neutrophils and IL-8 levels in nasal lavage fluid [32] and the levels of inflammatory cytokines produced by human airway epithelial cells [33]. The local inflammation caused by PM in the alveoli could further develop into a systemic inflammatory state [34], which is an essential event for many diseases.

In this study, SO_2_ was not associated with RDs in single-pollutant models. After adjusting for PM_2.5_, NO_2_ and CO in two-pollutant model, negative associations between SO_2_ and RDs were observed. Previous studies on exposure to SO_2_ and RDs have yielded mixed results. A time-series analysis in Jinan showed that an increase of 10 μg/m^3^ in SO_2_ was associated with 1.69% (95%CI, 1.56–1.83%) increase in daily non-accidental mortality rate [12]. Another recent study showed strong association between SO_2_ and outpatient visits for RDs in southeastern China [35]. In addition, several studies reported a null association [22, 36]. However, in line with the present study, another study found that outdoor SO_2_ significantly associated with the reduced risk of initial outpatient visits for tuberculosis, suggesting short-term protective effects of SO_2_ exposure on bacteria-induced pulmonary infections [37]. The inconsistency of conclusions may be explained by differences in compositions of air pollutants or individual sensitivity [35]. The effects of SO_2_ need to be confirmed in future large-scale population based studies.

In the present study, the majority of the outpatients were males and less than 45 years old. Literatures have shown that males have less mature lungs and relatively narrower airways compared with females [38, 39]. Besides, children have insufficient antioxidant defenses and weakened ability of scavenging exogenous toxicants [40]. Therefore, males and younger people may have a higher susceptibility for damage by exposure to air pollutants even when outdoor air pollutants concentrations are not high. These factors could possibly contribute to the large number of daily outpatients among males and younger people. However, in gender-specific analysis, the associations of PM_2.5_ and PM_10_ were significant both in males and females. In age-specific analysis, the magnitudes of the effect estimates of PMs were greater in older people, which indicated that with the concentrations of PMs increased, the number of older outpatients saw larger growth. This evidence suggested that older people should minimize their outdoor activities and pay more attention on personal protection when outdoor PMs concentrations are high. We found PM_2.5_ and PM_10_ were significantly associated with acute RDs only. One of the explanations might be that the daily count of outpatient visits for acute RDs was much larger than that for chronic RDs (702 for acute RDs vs. 50 for chronic RDs per day). Other unknown mechanisms underlying the associations of PM with acute and chronic RDs might exist and need to be further investigated.

The strengths of our study are noteworthy. First, compared to the majority of previous studies that focused on a selected population (such as children) or a specific diagnosis, our study was characterized by the diversity of RDs diagnoses, the representation of the general population, and long time span. Second, to reduce the selection bias as much as possible, we collected data from five hospitals in Jinan city. Third, the large sample size enabled us to examine the nonlinear association between air pollutant exposures and outpatient visits for RDs and to further investigate the effect modification by gender, age group and the type of RDs. Nevertheless, our study has some limitations. Our study focused on one city in China, so the results should be interpreted with caution for other city or population. Another limitation is that unknown or unmeasured confounders such as smoking status, vaccinations and so on may exist and contributed to the associations.

## Conclusions

In conclusion, the present study suggested that outdoor air pollution associated with increased risk of outpatient visits for RDs in China. PM_2.5_ and PM_10_ showed instant and continuous effects, while NO_2_, CO and O_3_ showed delayed effects on outpatient visits for RDs. Besides, PMs showed significant associations with acute RDs only. Ongoing efforts are required to better understand the adverse effects of outdoor air pollution on public health and to develop feasible preventive approaches.

## Additional file

Additional file 1: **Table S1** Percentage changes in outpatient visits for RDs associated with a 10 μg/m^3^ increase in concentration of each pollutant in using different lag structures in single-pollutant models. **Table S2** Percentage changes in outpatient visits for RDs associated with a 10 μg/m^3^ increase in concentration of PM_2.5_ across different gender and age groups. **Table S3** Percentage changes in outpatient visits for RDs associated with a 10 μg/m^3^ increase in concentration of PM_10_ across different gender and age groups. **Table S4** Percentage changes in outpatient visits for different type of RDs associated with a 10 μg/m^3^ increase in concentration of PM_2.5_ and PM_10_. (DOC 108 kb)

## Abbreviations

AIC: Akaike’s information criterion
CI: Confidence interval
CO: Carbon monoxide
DOW: Day of the week
GAM: Generalized additive model
Max: Maximum; Min: minimum
NO_2_: Nitrogen dioxides
O_3_: Ozone
P_25_: 25th percentile
P_50_: 50th percentile
P_75_: 75th percentile
PM: Particle matter
PM_10_: Particle matter≤10-μm in diameter
PM_2.5_: Particle matter≤2.5-μm in diameter
RDs: Respiratory diseases
SD: Standard deviation
SO_2_: Sulfur dioxide
