# Supplementary material for: The impact of outdoor air pollutants on outpatient visits for respiratory diseases during 2012–2016 in Jinan, China
Source: Respir Res. 2018 Dec 12;19:246. doi: 10.1186/s12931-018-0958-x (PMC6292059; doi:10.1186/s12931-018-0958-x)
Supplement: Supplementary file 1 — Table S1 Percentage changes in outpatient visits for RDs associated with a 10 μg/m3 increase in concentration of each pollutant in using different lag structures in single-pollutant models. Table S2 Percentage changes in outpatient visits for RDs associated with a 10 μg/m3 increase in concentration of PM2.5 across different gender and age groups. Table S3 Percentage changes in outpatient visits for RDs associated with a 10 μg/m3 increase in concentration of PM10 across different gender and age groups. Table S4 Percentage changes in outpatient visits for different type of RDs associated with a 10 μg/m3 increase in concentration of PM2.5 and PM10. (DOC 108 kb) [file 12931_2018_958_MOESM1_ESM.doc]

**Table S1** Percentage changes in outpatient visits for RDs associated with a 10 μg/m^3^ increase in concentration of each pollutant in using different lag structures in single-pollutant models

| **Lag day** | **PM_2.5_** | **PM_10_** | **SO_2_** | **NO_2_** | **CO** | **O_3_** |
| --- | --- | --- | --- | --- | --- | --- |
| Single-day lags | | | | | | |
| Lag0 | 0.159(0.035,0.284)* | 0.122(0.042,0.202)* | -0.155(-0.313,0.004) | 0.361(0.000,0.723)* | 0.010(-0.001,0.021) | 0.136(-0.079,0.353) |
| Lag1 | 0.139(0.032,0.247)* | 0.112(0.040,0.184)* | 0.017(-0.136,0.170) | **0.527(0.211,0.843)*** | **0.013(0.003,0.023)*** | 0.111(-0.068,0.290) |
| Lag2 | 0.133(0.035,0.233)* | 0.113(0.045,0.182)* | -0.020(-0.169,0.130) | 0.365(0.065,0.665)* | 0.008(-0.001,0.017) | 0.122(-0.039,0.282) |
| Lag3 | **0.168(0.072,0.265)*** | **0.149(0.082,0.215)*** | 0.021(-0.127,0.169) | 0.514(0.224,0.806)* | 0.013(0.004,0.021)* | 0.134(-0.024,0.292) |
| Lag4 | 0.119(0.023,0.215)* | 0.120(0.053,0.186)* | 0.066(-0.082,0.214) | 0.427(0.139,0.716)* | 0.009(0.000,0.018)* | **0.189(0.032,0.347)*** |
| Lag5 | 0.058(-0.038,0.155) | 0.080(0.013,0.147)* | 0.056(-0.091,0.204) | 0.312(0.024,0.602)* | 0.003(-0.006,0.012) | 0.148(-0.009,0.305) |
| Aggregated lags | | | | | | |
| Lag01 | 0.217(0.078,0.356)* | 0.165(0.075,0.256)* | -0.103(-0.298,0.092) | 0.660(0.255,1.066)* | 0.018(0.005,0.030)* | 0.211(-0.046,0.468) |
| Lag02 | 0.272(0.121,0.422)* | 0.213(0.114,0.312)* | -0.108(-0.335,0.119) | 0.787(0.346,1.231)* | 0.020(0.006,0.034)* | 0.265(-0.003,0.533) |
| Lag03 | 0.358(0.195,0.520)* | 0.287(0.179,0.394)* | -0.088(-0.345,0.169) | 1.031(0.554,1.510)* | 0.027(0.012,0.043)* | 0.324(0.044,0.606)* |
| Lag04 | 0.414(0.238,0.589)* | 0.346(0.229,0.463)* | -0.038(-0.323,0.248) | 1.203(0.695,1.714)* | 0.032(0.016,0.049)* | 0.425(0.128,0.723)* |
| Lag05 | **0.441(0.251,0.632) *** | **0.388(0.261,0.514)*** | 0.004(-0.308,0.316) | **1.310(0.770,1.852)*** | **0.034(0.016,0.051)*** | **0.499(0.183,0.815)*** |

^*^*P*<0.05; Bond, the maximum effect estimates; All analyses were adjusted for calendar time, the day of the week, daily temperature, humidity, air pressure and wind speed.

**Table S2** Percentage changes in outpatient visits for RDs associated with a 10 μg/m^3^ increase in concentration of PM_2.5_ across different gender and age groups

| Lag day | Total |  | Gender | |  | Age | | | | |
| --- | --- | --- | --- | --- | --- | --- | --- | --- | --- | --- |
|  |  |  | Male | Female |  | <18y | 18-44y | 45-64y | >64y | |
| Single-day lags |  |  |  |  |  |  |  |  |  | |
| Lag 0 | 0.159(0.035,0.284)* |  | 0.143(0.016,0.271)* | 0.184(0.045,0.324)* |  | **0.145(0.019,0.271)*** | 0.115(-0.036,0.266) | **0.281(0.072,0.491)*** | 0.189(-0.083,0.463) | |
| Lag 1 | 0.139(0.032,0.247)* |  | 0.141(0.031,0.251)* | 0.136(0.016,0.257)* |  | 0.102(-0.007,0.211) | 0.160(0.030,0.291)* | 0.168(-0.013,0.350) | 0.186(-0.049,0.422) | |
| Lag 2 | 0.133(0.035,0.233)* |  | 0.129(0.028,0.231)* | 0.140(0.029,0.251)* |  | 0.057(-0.043,0.157) | 0.187(0.067,0.307)* | 0.127(-0.041,0.294) | 0.243(0.025,0.461)* | |
| Lag 3 | **0.168(0.072,0.265)*** |  | **0.147(0.048,0.246)*** | **0.201(0.093,0.310)*** |  | 0.059(-0.038,0.157) | **0.203(0.086,0.319)*** | 0.234(0.071,0.398)* | **0.319(0.106,0.533)*** | |
| Lag 4 | 0.119(0.023,0.215)* |  | 0.109(0.011,0.208)* | 0.135(0.027,0.243)* |  | 0.016(-0.081,0.113) | 0.113(-0.004,0.230) | 0.252(0.090,0.415)* | 0.246(0.034,0.458)* | |
| Lag 5 | 0.058(-0.038,0.155) |  | 0.053(-0.045,0.152) | 0.066(-0.043,0.174) |  | -0.014(-0.111,0.084) | 0.034(-0.083,0.151) | 0.175(0.012,0.338)* | 0.153(-0.059,0.366) | |
| Aggregated lags | | | | | | | | | |  |
| Lag 01 | 0.217(0.078,0.356)* |  | 0.208(0.066,0.351)* | 0.230(0.074,0.386)* |  | 0.177(0.036,0.318)* | 0.207(0.038,0.376)* | 0.316(0.082,0.551)* | 0.274(-0.031,0.580) | |
| Lag 02 | 0.272(0.121,0.422)* |  | 0.262(0.108,0.416)* | 0.287(0.118,0.456)* |  | 0.181(0.029,0.334)* | 0.307(0.124,0.490)* | 0.343(0.090,0.597)* | 0.399(0.069,0.730)* | |
| Lag 03 | 0.358(0.195,0.520)* |  | 0.334(0.168,0.501)* | 0.394(0.212,0.577)* |  | **0.201(0.036,0.365)*** | 0.414(0.216,0.611)* | 0.466(0.192,0.741)* | 0.577(0.219,0.936)* | |
| Lag 04 | 0.414(0.238,0.589)* |  | 0.385(0.205,0.565)* | 0.459(0.262,0.656)* |  | 0.198(0.020,0.375)* | 0.462(0.249,0.676)* | 0.605(0.308,0.902)* | 0.705(0.318,1.094)* | |
| Lag 05 | **0.441(0.251,0.632)*** |  | **0.410(0.216,0.605)*** | **0.491(0.278,0.704)*** |  | 0.184(-0.008,0.376) | **0.474(0.243,0.705)*** | **0.705(0.384,1.027)*** | **0.788(0.368,1.209)*** | |

^*^*P*<0.05; Bond, the maximum effect estimates; All analyses were adjusted for calendar time, the day of the week, daily temperature, humidity, air pressure and wind speed.

**Table S3** Percentage changes in outpatient visits for RDs associated with a 10 μg/m^3^ increase in concentration of PM_10_ across different gender and age groups

| Lag day | Total |  | Gender | |  | Age | | | |
| --- | --- | --- | --- | --- | --- | --- | --- | --- | --- |
|  |  |  | Male | Female |  | <18y | 18-44y | 45-64y | >64y |
| Single-day lags |  |  |  |  |  |  |  |  |  |
| Lag 0 | 0.122(0.042,0.202)* |  | 0.117(0.034,0.199)* | 0.130(0.040,0.220)* |  | **0.087(0.005,0.169)*** | 0.105(0.008,0.202)* | 0.206(0.072,0.341)* | 0.180(0.003,0.357)* |
| Lag 1 | 0.112(0.040,0.184)* |  | 0.121(0.047,0.195)* | 0.098(0.017,0.179)* |  | 0.079(0.006,0.153)* | 0.134(0.047,0.221)* | 0.138(0.017,0.259)* | 0.135(-0.024,0.293) |
| Lag 2 | 0.113(0.045,0.182)* |  | 0.12(0.050,0.189)* | 0.104(0.027,0.180)* |  | 0.064(-0.005,0.133) | 0.163(0.081,0.245)* | 0.106(-0.009,0.221) | 0.144(-0.007,0.294) |
| Lag 3 | **0.149(0.082,0.215)*** |  | **0.145(0.076,0.213)*** | **0.156(0.081,0.230)*** |  | 0.083(0.016,0.151)* | **0.194(0.114,0.274)*** | 0.173(0.061,0.286)* | **0.186(0.038,0.335)*** |
| Lag 4 | 0.120(0.053,0.186)* |  | 0.113(0.045,0.181)* | 0.131(0.056,0.205)* |  | 0.049(-0.018,0.117) | 0.122(0.042,0.202)* | **0.214(0.102,0.325)*** | 0.183(0.035,0.330)* |
| Lag 5 | 0.080(0.013,0.147)* |  | 0.076(0.008,0.144)* | 0.086(0.011,0.161)* |  | 0.018(-0.050,0.086) | 0.066(-0.014,0.147) | 0.174(0.062,0.286)* | 0.161(0.013,0.309)* |
| Aggregated lags | | | | | | | | | |
| Lag 01 | 0.165(0.075,0.256)* |  | 0.169(0.077,0.262)* | 0.159(0.058,0.261)* |  | 0.118(0.026,0.210)* | 0.172(0.063,0.282)* | 0.239(0.087,0.390)* | 0.220(0.021,0.418)* |
| Lag 02 | 0.213(0.114,0.312)* |  | 0.220(0.119,0.322)* | 0.201(0.090,0.312)* |  | 0.139(0.039,0.240)* | 0.254(0.135,0.374)* | 0.266(0.100,0.432)* | 0.277(0.059,0.495)* |
| Lag 03 | 0.287(0.179,0.394)* |  | 0.291(0.181,0.401)* | 0.281(0.160,0.402)* |  | 0.178(0.069,0.288)* | 0.354(0.224,0.484)* | 0.349(0.168,0.531)* | 0.368(0.130,0.607)* |
| Lag 04 | 0.346(0.229,0.463)* |  | 0.345(0.226,0.465)* | 0.347(0.216,0.478)* |  | 0.199(0.080,0.318)* | 0.411(0.270,0.552)* | 0.464(0.267,0.661)* | 0.463(0.203,0.723)* |
| Lag 05 | **0.388(0.261,0.514)*** |  | **0.384(0.255,0.514)*** | **0.393(0.251,0.536)*** |  | **0.205(0.076,0.335)*** | **0.444(0.290,0.597)*** | **0.562(0.348,0.776)*** | **0.553(0.271,0.836)*** |

^*^*P*<0.05; Bond, the maximum effect estimates; All analyses were adjusted for calendar time, the day of the week, daily temperature, humidity, air pressure and wind speed.

**Table S4** Percentage changes in outpatient visits for different type of RDs associated with a 10 μg/m^3^ increase in concentration of PM_2.5_ and PM_10_

| Lag day | PM_2.5_ | | |  | PM_10_ | | |
| --- | --- | --- | --- | --- | --- | --- | --- |
|  | Total RDs | Acute RDs | Chronic RDs |  | Total RDs | Acute RDs | Chronic RDs |
| Single-day lags | | | | | | | |
| Lag 0 | 0.159(0.035,0.284)* | 0.150(0.029,0.271)* | 0.262(-0.149,0.675) |  | 0.122(0.042,0.202)* | 0.116(0.038,0.194)* | 0.199(-0.067,0.465) |
| Lag 1 | 0.139(0.032,0.247)* | 0.139(0.034,0.243)* | 0.153(-0.199,0.507) |  | 0.112(0.040,0.184)* | 0.113(0.043,0.183)* | 0.082(-0.154,0.320) |
| Lag 2 | 0.133(0.035,0.233)* | 0.140(0.044,0.236)* | -0.012(-0.341,0.318) |  | 0.113(0.045,0.182)* | 0.122(0.056,0.188)* | -0.066(-0.293,0.161) |
| Lag 3 | **0.168(0.072,0.265)*** | **0.176(0.083,0.270)*** | 0.049(-0.273,0.373) |  | **0.149(0.082,0.215)*** | **0.159(0.095,0.224)*** | -0.030(-0.253,0.194) |
| Lag 4 | 0.119(0.023,0.215)* | 0.112(0.019,0.205)* | 0.237(-0.081,0.557) |  | 0.120(0.053,0.186)* | 0.118(0.054,0.183)* | 0.129(-0.093,0.351) |
| Lag 5 | 0.058(-0.038,0.155) | 0.055(-0.038,0.149) | 0.069(-0.25,0.388) |  | 0.080(0.013,0.147)* | 0.076(0.011,0.141)* | 0.096(-0.127,0.319) |
| Aggregated lags | | | | | | | |
| Lag 01 | 0.217(0.078,0.356)* | 0.210(0.075,0.346)* | 0.292(-0.166,0.752) |  | 0.165(0.075,0.256)* | 0.163(0.075,0.250)* | 0.190(-0.108,0.489) |
| Lag 02 | 0.272(0.121,0.422)* | 0.272(0.126,0.418)* | 0.218(-0.277,0.716) |  | 0.213(0.114,0.312)* | 0.217(0.121,0.313)* | 0.107(-0.221,0.435) |
| Lag 03 | 0.358(0.195,0.520)* | 0.364(0.206,0.521)* | 0.226(-0.311,0.766) |  | 0.287(0.179,0.394)* | 0.297(0.193,0.402)* | 0.076(-0.281,0.435) |
| Lag 04 | 0.414(0.238,0.589)* | 0.414(0.244,0.585)* | 0.366(-0.212,0.948) |  | 0.346(0.229,0.463)* | 0.355(0.242,0.468)* | 0.151(-0.238,0.540) |
| Lag 05 | **0.441(0.251,0.632)*** | **0.440(0.256,0.625)*** | 0.398(-0.225,1.026) |  | **0.388(0.261,0.514)*** | **0.394(0.272,0.517)*** | 0.205(-0.217,0.628) |

^*^*P*<0.05; Bond, the maximum effect estimates; All analyses were adjusted for calendar time, the day of the week, daily temperature, humidity, air pressure and wind speed.
